# Dyadic synchrony in deaf mothers and hearing infants

**DOI:** 10.1093/jdsade/enag005

**Published:** 2026-02-19

**Authors:** Harriet Bowden-Howl, Rudi Dallos, Laura Goldberg, Evelyne Mercure

**Affiliations:** University of Plymouth, School of Psychology, Portland Square, University of Plymouth, Drake Circus, Plymouth, PL4 8AA, United Kingdom; University of Plymouth, School of Psychology, Portland Square, University of Plymouth, Drake Circus, Plymouth, PL4 8AA, United Kingdom; University College London, Institute of Cognitive Neuroscience, Alexandra House, 17 Queen Square, London, WC1N 3AZ, United Kingdom; University College London, Institute of Cognitive Neuroscience, Alexandra House, 17 Queen Square, London, WC1N 3AZ, United Kingdom; Goldsmiths, University of London, School of Mind, Body and Society, 8 Lewisham Way, London, SE14 6NW, United Kingdom

## Abstract

There is mounting evidence that maternal sensitivity contributes to optimal child development, but little is known about dyads including deaf mothers and their hearing infants. Deaf adults experience a range of adversities in their role as a parent, and it is unclear how these experiences influence early interactions with their child. Videos of 28 deaf mothers playing with their four-to-eight-month-old hearing infants were coded using the CARE-Index. Dyadic synchrony of most dyads were found to be sensitive or adequate. However, 21% of dyads were likely to benefit from education and/or intervention to increase sensitive interactions. Compared to the rest of the dyads, they displayed lower levels of maternal sensitivity and infant cooperativeness, as well as higher levels of maternal unresponsiveness and infant passivity. Results were influenced by socioeconomic status and potentially by vocal and social touch behaviour. Methodological, theoretical and clinical considerations are offered for professionals working with deaf mothers and their hearing infants.

90% of deaf parents have hearing children (Bishop & Hicks, 2005; Preston, 1995) but there is a paucity of literature that explores the patterns of interaction between deaf mothers and their hearing infants. Hearing infants who grow up with deaf caregivers are likely to experience differences in their childhood to hearing infants who have hearing caregivers; however, few studies have explored the emotional impact of these experiences.

This paper aims to view maternal deafness not as a pathological issue, but rather as a difference in socio-cultural experience for the child. The first author sees herself as part of the deaf community; born profoundly deaf and raised by hearing parents. Deaf people in the UK are more likely to have experienced poor educational attainment as a child compared to their hearing peers (National Deaf Children’s Society, 2025), which may account for their lower employment rate, their lower average household income, and the overrepresentation of deaf adults in lower status, lower paid posts compared to hearing adults (Shield, 2019). The prevalence of mental health problems is also higher within the deaf than the hearing population (Fellinger et al., 2012). Deaf people suffer higher rates of depression compared to the rest of the population (24% compared with 12%), and common mental health problems are likely to be underreported (The Deaf Health Charity SignHealth, 2014).

When deaf mothers who have hearing children were asked their views on family life, they felt that information was scarce and support from services, such as parenting classes, were practically unavailable to them (Allsop & Kyle, 1997; St Clair et al., 2025). In a recent qualitative study, deaf parents revealed significant difficulties accessing parenting information and support, experiencing prejudice and discrimination in their parenting journey, and having to navigate extra stress and expend more energy to overcome these barriers (St Clair et al., 2025). Allsop and Kyle (1997) comment on the lower socio-economic position of deaf households compared to hearing households. They also explore deaf parents’ worries about having a hearing child, for example, being aware that they needed specific guidance around optimal communication and their child’s development of language. Therefore, deaf parents experience a range of adversities which need to be borne in mind when researching and working with this population. Singleton and Tittle (2000) summarize the differences that may be experienced when deaf parents bring up hearing children, including culture, communication patterns, and parenting issues. They state that “Deaf individuals born to hearing parents do not automatically acquire the hearing culture of their parents, nor do they share hearing culture with their own hearing children”. To date, the limited research that has been conducted in this area has largely focused on communicative strategies, speech, and language development rather than the emotional wellbeing of infants (Capirci et al., 2002).

Studying the quality of the interaction between parents and their children helps us understand what will shape the infant’s development into an adult equipped with different ways of relating to others. The relationship is the most important aspect of the parent–child interaction (Bowlby, 1969) because an available and understanding parent will support the child to be able to play more creatively, mentalize from their own and others’ perspectives, develop a sense of their own agency and regulate their own emotion (Music, 2024). They will also develop positive expectations about future relationships, develop trust in others, and gain self-reliance (Golding, 2007).

Ainsworth (1969) succinctly defined sensitivity as “the mother’s ability to perceive and to interpret accurately the signals and communications implicit in her infant’s behaviour, and given this understanding, to respond to them appropriately and promptly”. Van den Boom (1997), in agreement with Ainsworth, considered the definition of sensitivity and rather than seeing it as a construct that belongs only to the parents, clarified that sensitivity is about interaction and must refer to both individuals in the dyad. This leads to the idea of dyadic synchrony. A systematic review conducted by Leclère et al. (2014) demonstrated a link between synchrony and attachment as well as child cognitive and behavioural development. Farnfield et al. (2010) refers to dyadic synchrony as the “dance” between attachment figure and child. Adult sensitivity is defined by Crittenden (2010) as “any pattern of behavior [in play] that pleases the infant and increases the infant’s comfort and attentiveness and reduces its distress and disengagement”.

The CARE-Index (Crittenden, 2010) is an instrument that has been developed building on Ainsworth’s conceptualisation. It assesses the developing relationship and interactional characteristics between a caregiver and an infant and provides information about dyadic synchrony. According to Farnfield et al. (2010) the CARE-Index is the most valid tool for researching parenting interactions with low-risk populations, different social classes, and cultural backgrounds. The global dyadic synchrony scale (as named in the CARE-Index) is also related to maternal sensitivity (Crittenden, 2010). Being assessed as having a sensitive dyadic synchrony indicates that there was mutual delight and shared positive affect within the dyad where a smooth pleasing interaction could be seen between the two active participants.

While there have been studies on language and communication between deaf parents and their children, fewer studies have focused on the development of the emotional relationship in these dyads. In terms of the dyadic relationship, Jones (1996) used the Nursing Child Assessment Teaching Scale to study parent–child interactions (parental sensitivity to cues, response to the child’s distress, social–emotional and cognitive growth fostering) comparing six deaf mothers and six hearing mothers. No significant differences were observed between the two groups, inferring that mothers and their infants adapt to each other’s unique characteristics. However, the small sample size raises questions as to whether these findings hold sufficient statistical power. Despite these non-significant findings, five of the six deaf mothers did not actively respond to their child when they were in distress and seemingly waited for their child to return their attention to the task by themselves. In addition to this, Jones (1996) stated that none of the deaf mothers touched their infants in an affectionate manner (patting or caressing) during the task. One potential explanation for this finding may be that the deaf mothers’ hands were occupied by the use of sign language and that affection may have been communicated through non-tactile means. This hypothesis remains to be formally tested.

In contrast, Meadow-Orlans and Spencer (1996) found that deaf mothers with hearing infants were rated as more sensitive than hearing mothers with deaf infants when they were 12 months old. However, when the infants were 18 months old, they found deaf mothers’ sensitivity with their hearing infants were significantly less likely to be rated positively when compared to either of the matched hearing status dyads. They suggested that being a deaf mother to a hearing infant and feeling different to the infant may trigger feelings of incompetence in the mother and affect interactions.

Finally, in a study designed to explore at-risk differences between groups; deaf mothers were compared to middle class mothers, neglecting mothers, abusing mothers, “intellectually disabled” mothers and low-income mothers (Crittenden & Bonvillian, 1984). The deaf mothers were deemed to be less consistently sensitive in comparison to the middle-class hearing sample, but more sensitive than the other groups. Deaf mothers were less likely than hearing middle-class mothers to speak to their infants and if they did, their vocal expression was more likely to be flat. They were also less likely to take turns vocalising or playing with their infant; and more likely to interfere in the child’s play potentially because of a misinterpretation of infant signals. However, the authors noted that the deaf mothers attempted to compensate for their deafness through clarity and consistency of their commands towards their infants and if the interaction was not dependent on vocalized cues, they were deemed to be very sensitive to their infants. The authors explained that seven of the ten deaf families communicated primarily through American Sign Language, however, the study did not discuss the use of visual communication to respond to their infants’ needs.

## Aims and hypotheses

The general aim of the present study was to explore emotional interactions between deaf mothers and their hearing infants. First, the clinical implications of these emotional interactions were considered by categorising their dyadic synchrony scores into four categories as directed by the CARE-Index: sensitive, adequate, intervention and at-risk. From these four categories, dyads labelled as intervention or at-risk are likely to benefit from education and/or intervention, while sensitive or adequate dyads are considered to have good interactions. The second aim of the present study was to characterize the interaction patterns in dyads likely to benefit from education and/or intervention compared to dyads with good interactions in terms of maternal descriptor scales (sensitivity, control, and unresponsiveness) and infant descriptor scales (cooperation, compulsivity, difficult behaviour, and passivity). Finally, to clarify the characteristics of dyads most likely to benefit from extra support, we assessed the influence of demographic and socioeconomic factors on each of these maternal and infant descriptors that differed significantly between groups.

## Methodology

### Participants

Data is presented for 28 deaf mothers interacting with their hearing infants between 4 and 8 months. Deaf mothers used BSL as their preferred mode of communication, and self-identified as profoundly deaf (n = 18), severely deaf (n = 5), deaf (n = 1) or hard of hearing (n = 2). Fifteen mothers used hearing aids, 4 used cochlear implants and 7 used no hearing assistive technology. Two mothers did not respond to questions about their hearing levels and use of hearing assistive technology. Table 1 provides a complete description of the sample’s demographic characteristics. Data from a further 3 dyads was excluded for the following reasons: withdrawal from the study (n = 1), insufficient video length (n = 1), the returning of a family with a younger sibling (n = 1). We have not included data that was collected from 62 dyads with hearing mothers and their hearing infants as the focus of the paper is on diversity within the group of dyads with a deaf mother.

**Table 1:** Demographic characteristics of the sample.

Characteristics	*M (SD)*
Infants’ Age Infants’ Gender	6.4 (1.1) 57% female
Maternal Age	32.6 (4.2)
% Ethnicity *White British* *White Other* *British* *British Asian* *Black British* *Other*	59% 22% 7.5% 7.5% 0% 4%
% Biological mother	96%
% Employment Status *Full-time* *Part-time* *In education* *Unemployed* *Part-time and in education* *Full-time and in education*	36% 28% 7% 18% 7% 4%
% Educational Level *GCSES* *A-Levels* *Degree/HND* *Postgraduate degree/Doctorate*	14% 18% 36% 33%
% Presence of depression in the family Household income <£20,000 £20,000–29,999 £30,000–39,999 £40,000–59,999 £60,000–79,999 £80,000–99,999 £100,000–149,999 >£149,999	36%
	16% 13% 25% 8% 17% 21% 0% 0%
Infant’s dominant language modality Sign language 50/50 Sign/Spoken Spoken language	64% 7% 29%

*Note*. M = Mean score; SD = standard deviation; GCSES = General Certificate of Secondary Education*;* HND = Higher National Diploma.

Data was collected as part of a larger study, which included a filmed interaction between deaf and hearing mothers with their hearing infants as well as neuroimaging and eye-tracking studies discussed elsewhere (Mercure et al., 2018, 2019, 2020, 2025). Deaf parents were recruited through social media and parenting groups and websites specifically aimed at the deaf community. Before data collection began, this project received ethical approval from the research ethics committees of UCL, and Birkbeck. In addition, the data analyses presented in the present article received ethical approval from the research ethics committee of University of Plymouth. Written informed consent was obtained from all parents after the study was explained by a member of the research team who was fluent in English or BSL as preferred by each family.

### Procedure

The recordings of parent–child interaction took place at the Birkbeck Babylab, where a room was especially designed for unobtrusive recording of activities. Each parent was asked to “play with your child like you would do at home” and the dyads were filmed for approximately 12 minutes. The first 6 minutes was with the infant in a rocker chair with no toys present and then the parent was asked to take the infant out of the rocker and a standard collection of seven toys was made available. Only the part of the video with the infant out of the rocker, on the floor with toys available to them was coded in this study.

### The CARE-Index

Mother-infant dyads were coded using the CARE-Index. Coding commenced when the mother took the infant out of the rocker and continued for five minutes. The CARE-Index assesses sensitivity in seven ways: facial expression, verbal expression, position, and body contact [within the dyad], affection, turn-taking contingencies, control, and choice of activity [temporal contingencies]. Maternal interactional sensitivity is considered alongside maternal controlling interactional behaviour and unresponsive interactional behaviour. Infant signifier behaviours are cooperative, difficult, compulsive, and passive.

Under the CARE-Index guidance, spoken utterances by parents are not coded for their linguistic content, but for their vocal tone/affect and contingency. Higher sensitivity scores are given to utterances with a warm, positive and engaged tone of voice; as well as for parental utterances that closely follows a child’s vocalisation. Sign language utterances were coded in a similar way with quality determined by facial expression, body language, eye gaze and visual attunement, and contingency as the timing of the parent’s sign, gesture, or behaviour in relation to the child’s. A parental response that follows a child’s vocalisation, sign or gesture was rated as sensitive, while a delayed or absent parental response indicated unresponsiveness or a rupture in the synchrony of the interaction.

The coding of the maternal and infant interaction is also considered within the “global scale of dyadic synchrony” where scores range from 0 to 14; 0–4 indicates that that the dyadic-interaction is at-risk, 5–6 is at the intervention level, 7–10 is at an adequate level and 11–14 is at a sensitive level. These scales, especially the dyadic synchrony scales have been found to predict attachment style (e.g., Fuertes et al., 2009; Fuertes et al., 2016; Udry-Jorgensen et al., 2011). Videos were coded by one of two CARE-Index trained raters or both (28%) and interrater intraclass correlation coefficients for maternal sensitivity, control and unresponsiveness were: .80, .61, and .71, respectively, and for the infant descriptor scales; infant cooperativeness, compulsivity, difficultness, and passivity: .88, .85, .92, and .71, respectively.

Prior to undertaking the analysis, both the first and second authors completed CARE-Index Infant training and achieved good reliability which was externally validated. The recordings were allocated between the authors to ensure that none of the dyads were known personally to the first author (due to the small size of the overall population). Inter-rater reliability was tested for 28% of the dyads and any discrepancies were discussed. Discrepancies found were predominately regarding the vocalisations and touch in the observed deaf mother-infant dyads. Crittenden (personal communication, January 19, 2017) emphasized that for pre-verbal infants, the content of the words does not matter but the non-verbal qualities are important; if mothers do not use speech, speak in a flat tone, speak loudly or out of synchrony with the child’s actions it is important to score this from the infants’ perspective. So, any mis-articulations and unusual tones that deaf mothers produced were coded accordingly. An additional 14% of the two coders’ recordings were rated independently by an experienced coder who was a CARE-Index trainer. Following substantial discussions about any differences in scoring between the coders’, agreed adjustments were applied to the other recordings before interrater reliability was computed.

## Results

Dyadic synchrony scores were very diverse within the group ranging from 3 to 14 with a mean of 7.96 and a standard deviation of 2.58 (from a possible range of 0 to 14, where a higher number indicates higher level of dyadic synchrony). Dyads were classified under four categories of dyadic synchrony as directed by the CARE-Index to understand any clinical implications that may raise for professionals working with this population: sensitive, adequate, intervention, and at-risk. Of the 28 dyads, 5 were rated as sensitive, 17 as adequate, 5 as intervention, and 1 as at-risk. According to the CARE-Index manual, scores of 6 or below indicate dyads who may benefit from parental education and/or intervention (intervention or at-risk). 21% of the dyads in the current group reached this education/intervention threshold, while 79% of dyads were considered as having good enough interactions.

**Figure 1:**
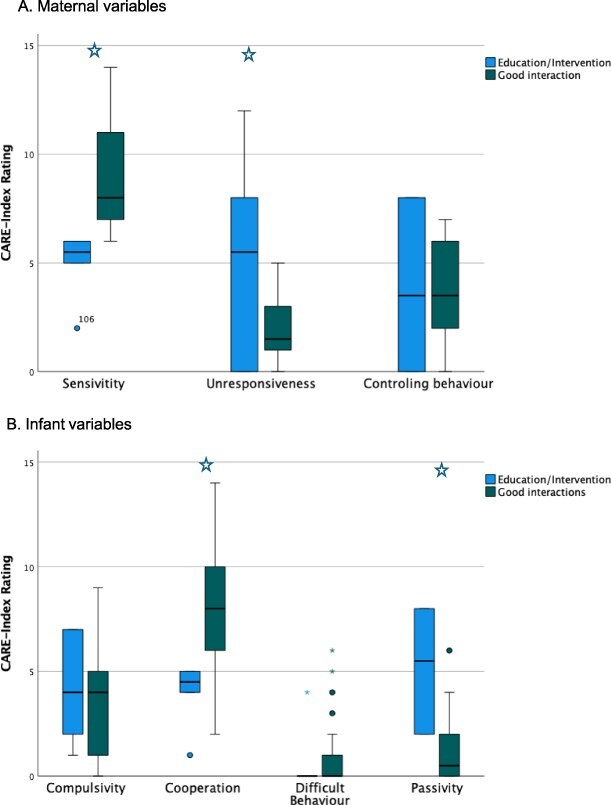
Comparison of CARE-Index ratings between dyads likely to benefit from intervention/education and dyads with good enough interactions for (A) maternal variables and (B) infant variables. Error bars display standard error. Light blue stars represent significant group effects.

To better characterize interaction patterns in dyads categorized as likely to benefit from education/intervention versus those categorized as good enough, maternal and infant variables were compared across these two global groups in a multivariate analysis of variance. The global group had a significant effect on the maternal and infant variables [F(7, 52) = 3.35; *p* = .005; = .31] (see Figure 1). Dyads in the education/intervention group were lower in maternal sensitivity [F(1) = 18.36; *p* < .001; = .24], and infant cooperation [F(1, 58) = 13.03; *p* = .001; = .18], but higher on maternal unresponsiveness [F(1, 58) = 15.04; *p* < .001; = .21], and infant passivity [F(1, 58) = 13.49; *p* = .001; = .19]. Maternal controlling behaviour [F(1, 58) = .39; *p* = .538; = .01], infant compulsivity [F(1, 58) = 1.08; *p* = .304; = .02] and infant difficult behaviour [F(1, 58) = .33; *p* = .571; = .01] did not differ between groups.

A series of forward regression models were performed to better understand the impact of demographic variables on the maternal and infant variables that differed between the two global groups. Each model tested the impact of maternal education, maternal age, household income and dominant language modality. For maternal sensitivity and infant cooperation, no factor had a significant impact on the dependent variable and no model was therefore built. For maternal unresponsiveness, the model was significant and explained 25.6% of the variance [R^2^ = .256; F(1,23) = 7.58; *p* = .012]. Household income was the only significant predictor entered in the model [B = −.802; t = −2.76; *p* = .012]. For infant passivity, the model was also significant, explaining 16.7% of the variance [R^2^ = .167; F(1,23) = 4.40; *p* = .048]. Like for maternal unresponsiveness, household income was the only demographic predictor of infant passivity [B = −.579; t = −2.10; *p* = .048]. Dyads of low household income were more likely to display an interaction style characterized by higher levels of maternal unresponsiveness and infant passivity.

## Discussion

This study aimed to further expand knowledge in the limited literature regarding how deaf mothers interact with their hearing infants. Despite the challenges that deaf mothers face, the synchrony of 79% of dyads was found to be sensitive or adequate. This may be considered as a surprising finding by some given how important spoken language is perceived to be to parent—child interactions in mainstream literature focused on hearing dyads. This data is a useful demonstration that sensitive parent–child interactions can also exist in the visuo-tactile channel, and in dyads with deaf parents. In the present study, 5 dyads with deaf mothers and their hearing infants were rated as sensitive and deemed to have a smooth, pleasing interaction with playful elements and shared positive affect. 17 dyads with deaf mothers were rated to be “adequate,” i.e., quite satisfactory play, where no significant problem was observed but some noticeable periods of desynchrony. All these dyads are considered to have good enough interaction that would not require parental education or intervention. Interestingly, the child’s dominant language of exposure (i.e., English or BSL) did not influence any of the maternal or infant variables, suggesting that sensitive interactions can be achieved by deaf mothers using any language modality.

However, 21% of dyads were found to be likely to benefit from education and/or intervention. Of these, 5 dyads were at intervention level, which means that they displayed a lack of empathy or limited playfulness, but no evidence of hostility. One dyad was rated as being at-risk, which is usually characterized by a clear lack of empathy, some insufficient or unsuccessful attempt to respond to the infant and a lack of playfulness. In comparison with dyads displaying good enough interactions, these six dyads were lower in maternal sensitivity and infant cooperativeness. Maternal sensitivity is defined as “any pattern of behaviour [in play] that pleases the infant and increases the infant’s comfort and attentiveness and reduces its distress and disengagement” (Crittenden & Bonvillian, 1984). To attain a high dyadic synchrony, the CARE-Index requires adults to be attentive and alert to the baby’s mood and the situation. Cooperative infants are generally attentive to the activity and show no avoidance of eye contact with the mother, whilst demonstrating a range of facial expressions. While most dyads with deaf mothers displayed interaction characterized by good levels of maternal sensitivity and infant cooperativeness, the six dyads lowest in dyadic synchrony were rated significantly lower on these variables. Interestingly, this pattern was not related to any demographic variable including maternal age, maternal education, household income and the child’s dominant language modality.

These six dyads likely to benefit from education/intervention were also rated higher than other dyads in maternal unresponsiveness and infant passivity. Mothers rated as unresponsive are likely to demonstrate a lower level of contingent responses to their infant. They may demonstrate low involvement in the interaction or present intermittent or unpredictable positive responses to the infant’s behaviour. Infants rated as passive are likely to show low levels of arousal and mood, low level of engagement and awareness of the play situation. They can display few vocalisations, few instances of eye contact or smiles, with a floppy hypotonic body. In the present study, there was also a significant relationship between these variables and household income: this pattern of unresponsive/passive interactions tended to be more represented in families of lower income. While income was the only demographic factor of significant effect, it is important to note that maternal education tended to have a similar impact on the maternal unresponsiveness at close to significant level (p = .056). Mothers with lower levels of formal education also tended to display more unresponsive behaviour than mothers with higher levels of formal education. Dyadic synchrony is known to be robustly affected by socioeconomic stress and risk (Crittenden & Bonvillian, 1984; Firk et al., 2018; Sturge-Apple et al., 2017). Unfortunately, socioeconomic disparities between deaf and hearing parents are likely to exist as deaf adults in the United Kingdom have lower household income on average and they are overrepresented in lower status, lower paid posts compared to hearing adults (Shield, 2019). The results of the present study suggest that this socioeconomic imbalance could put hearing children of deaf parents at risk of less favourable parent–child interaction. Further research would be required to clarify how socioeconomic status interact with maternal hearing status in the development of dyadic synchrony.

### Factors to consider when coding dyadic synchrony in dyads with a deaf parent

The CARE-Index was designed by hearing professionals with hearing parents of hearing children in mind. There are a few points to consider in order to sensitively measure dyadic synchrony in dyads with a deaf parent.

First of all, it is important to consider how maternal vocalisations by deaf mothers contributes to their emotional interaction ratings. Due to deaf mothers not being able to hear when growing up, their speech may take on differential patterns to hearing people. Some deaf people may have speech with inflections and intonations, whilst others are more likely to be monotone. Some deaf people do not use speech at all, relying only on British Sign Language and others cannot hear their own voices and therefore this affects volume control. When coding the CARE-Index, the vocal expression subscale captured how the mothers were using their voices to interact with their infants. For some, this immediately placed them at a disadvantage because their tone of voice was deemed to be harsh, monotone, too loud and/or unmodulated. However, it was deemed by Crittenden (personal communications) that deaf mothers could still be sensitive overall using their touch, movement and facial expression, even if their vocal expression was not. The CARE-Index development was based on the wider hearing population. Differences were found between the two coders (one of whom was deaf and the other hearing) of the CARE-Index in this study when discussing the interrater reliability, in relation to the deaf mothers. For example, discussions focused on attempting to understand if the deaf mothers were appropriately responsive to their infants, especially when considering touch and vocal expression. It was felt that differences may have been highlighted potentially because high dyadic synchrony may look different for deaf mothers and hearing mothers. For example, with the first author being deaf meant she had a greater understanding of how deaf people manage interactions and how sensitive interaction can be achieved. The second author, being non-deaf, could hear the dyads but placed more emphasis on the vocal expression scale as the more salient factor which influenced his overall experience of the dyads e.g., categorising them as more distant or controlling.

Another factor to consider is how body contact is used within the deaf community. When utilising the CARE-Index scoring, some body contact is considered to create a general physical wariness and/or instances of infant distress or discomfort in reaction to adult’s behaviour (Crittenden, 2010), as the infant is being made to comply physically with adult demands. However, visual communication strategies or sign language can also infer physical touch which can be used to gain and maintain attention from infants. Koester, Brooks and Traci (2000) found that the intense use of touch was evident within deaf mother—hearing infant dyads, as a mean to regain the infant’s visual attention. According to the authors, deaf mothers responded to the infant’s hand and arm movements in a similar way as hearing parents respond to an infant’s babbling; by “imitating, providing models, and gradually shaping these into word approximations” (Koester et al., 2000). Deaf mothers employed visual/non-vocal communication strategies more frequently than hearing mothers (Koester & Lahti-Harper, 2010). If deaf mothers noticed that their infants ignored/rejected them, they may use more non-vocal means to gain or maintain their infant’s attention compared to hearing mothers. This may be perceived as more controlling on a coding system such as CARE-Index. Hearing parents can speak to get their child’s attention without them having to look, touch or even be proximal. However, deaf parents are likely to need to check that their child has heard them; for example, by moving them. This may give the impression that they are controlling, especially if this is not combined with a kind facial expression or a smile. Rea et al. (1988) found deaf mothers to be comparable to hearing mothers in how they interacted with their infants and ascertained that there was no need for concern when considering the impact of maternal deafness on infant development. They concluded that touch may have been used as a substitute for vocalisation in getting the child’s attention or in forming the child’s hand into a sign. The CARE-Index aimed to explore subtle nuances in how parents interact with their infants as well as taking into consideration cultural differences as not every parent will respond to their child in a similar way. Some level of disagreement between raters was observed in the present study in terms of how maternal control was coded. It may be that raters with more experience of communication with deaf people view body contact more positively than hearing raters with limited contact with the deaf community. The CARE-Index manual encourages coders to apply coding similarly across cultures and if there is a difference, the meaning should be explored from a cultural perspective. This is what this paper is aiming to achieve.

Yet another factor that could have influenced the ratings of dyadic synchrony in this study is that deaf mothers are also potentially underreporting their mental health difficulties (The Deaf Health Charity SignHealth, 2014). This will inevitably impact on their ability to provide emotionally attuned and sensitive care for their children. In the present study, data collection was scarce in this area. Participants were asked if there was a history of depression in the family, but the data does not specify which family member this referred to. If it was the deaf mother herself or a close relative, this may well have shaped how the deaf mothers are feeling and interacting with their infants—this would have clarified whether mothers struggling with depression are more unresponsive, and their infants more passive in their behaviours. The World Health Organisation (2022) reported how young infants are likely to be affected by mothers who have mental illnesses. If the illness is severe or prolonged, it can hamper the mother-infant attachment and infant care. Unfortunately, we do not know about other forms of mental health issues in the present study, as they were not reported. It is worth noting that stress and grief experienced by hearing parents when finding out their infant is deaf can leave parents less emotionally available towards their child’s needs (Quittner et al., 2010; Vaccari & Marschark, 1997). Therefore, deaf children have a higher risk of developing an insecure attachment (Weisel & Kamara, 2005). Those deaf children then grow up to potentially become parents to hearing children who then in turn may experience difficulties in their interactions with their infants.

Finally, it is also important to consider the environment in which the parent—child interaction session took place. In the present study, parent and infants were filmed as part of a larger study taking place at the Birkbeck Babylab. Despite the presence of deaf individuals in the research team during data collection, it is possible that deaf parents felt judged or pressured to perform in a certain way when they came to participate in the present study in a predominantly hearing environment. This could have led some of the deaf mothers to display passive behaviour, for example. Of course, the situation of being filmed in a novel environment could influence the behaviour of any dyad, but a perceived clash between a predominantly hearing environment (the university) and a parent’s deaf culture, could have amplified this effect for dyads with a deaf mother. It would be important to clarify these findings by studying dyads interacting in their own environment after being contacted by a team of predominately deaf researchers.

### Considerations for intervention and conclusions

Despite the obvious challenges that deaf mothers face, dyadic synchrony of most deaf mothers and hearing infants was found to be sensitive or adequate. However, about a fifth of the dyads were found to have interaction patterns that suggest they could benefit from parental education or intervention. These dyads tended to have interaction patterns characterized by low maternal sensitivity, and low infant cooperation, while also showing high maternal unresponsiveness and high infant passivity.

There is a clear dilemma in the interpretation of these findings. On one hand, one must keep in mind that the tools used were designed for hearing dyads and that sensitive interaction may take different forms in the context of maternal deafness. This study aimed to be mindful of aspects of Deaf culture and sign language communication that could have influenced the patterns of interaction in dyads with a deaf mother. However, on the other hand, there is a possibility of minimising the risks faced by some of the dyads, limiting the importance of support for dyads with deaf mothers who may benefit from it. Whilst most deaf mothers were believed to be able to meet their infants’ needs in an adequate or sensitive manner, it is worth considering what professionals can offer deaf mothers who may struggle with this.

Observations of dyadic interactions may include clear unresolved problems, limited playfulness but no evidence of hostility or lack of empathy. Video feedback would be highly useful (e.g., Video Interactive Guidance or Video-feedback intervention to promote positive parenting and sensitive discipline) to promote attunement. This visual approach is well adapted to the reality of deaf mothers, particularly if feedback was delivered in British Sign Language for parents using this language modality. Parenting classes continue to be inaccessible to most deaf parents (St Clair et al., 2025); and the present study suggests that increased access to parenting support in sign languages would be of great benefit to deaf parents. Parent-infant psychotherapy is another recommendation for dyads with low dyadic synchrony. Again, this is currently inaccessible to most sign language using parents. It is important to seek out deaf clinicians or clinicians who can sign. At the very least an experienced and registered sign language interpreter should be provided for any psychotherapy session, and indeed any other interventions and parenting groups. Early intervention will be of benefit to dyads who are deemed “at-risk” due to a clear lack of empathy, responsiveness or playful quality, or a failure to soothe infants’ distressed state. Accessible individual relational therapy for the parent is recommended before involving the infant.

Exploring other factors, such as mental health and household income, would further our understanding of why there were dyads struggling more with sensitive interactions. It is interesting to consider how Deaf culture and the experiences of deaf mothers living within a hearing society can impact on their sensitivity towards their infant. Deaf mothers have a hard challenge of preparing their infants to enter a hearing world which may be quite a different culture to what they know. Despite these challenges, the present study demonstrated that most dyads with a deaf mother and their hearing infant achieved a level of dyadic synchrony qualified as sensitive or adequate using tools designed to assess hearing dyads.
